# Gut Check: Gastrointestinal Tuberculosis as a Potential Mimic of Another Disease

**DOI:** 10.7759/cureus.102111

**Published:** 2026-01-22

**Authors:** Rhett L Harmon, Mark Baniqued, Soonwook Hong, Hongying Tan, Thomas Kovacs, Shaun Chandna

**Affiliations:** 1 Internal Medicine, Olive View University of California Los Angeles Medical Center, Sylmar, USA; 2 Gastroenterology, University of California Los Angeles David Geffen School of Medicine, Los Angeles, USA; 3 Pathology, Olive View University of California Los Angeles Medical Center, Sylmar, USA; 4 Gastroenterology, Olive View University of California Los Angeles Medical Center, Sylmar, USA

**Keywords:** ascites, extrapulmonary tuberculosis, gastrointestinal tuberculosis, inflammatory bowel disease, terminal ileitis

## Abstract

Gastrointestinal tuberculosis (GI TB) is an uncommon form of extrapulmonary TB (EPTB). While it is more prevalent in endemic regions, its incidence is rising in developed countries due to factors such as immigration, HIV infection, and immunosuppressive therapy. We present a case of a 34-year-old woman with abdominal pain, nausea, and unintentional weight loss who was found to have imaging evidence of terminal ileal and colonic wall thickening, peritoneal thickening, and ascites, and was ultimately diagnosed with gastrointestinal TB after an endoscopic biopsy demonstrated noncaseating granulomatous ileitis, a cecal ulcer, and a positive GeneXpert *Mycobacterium tuberculosis* polymerase chain reaction (MTB-PCR) result in sputum.

This report highlights an unusual presentation characterized by a nonproductive cough and the absence of typical risk factors or manifestations of pulmonary TB. It underscores the importance of considering TB in the broad differential diagnosis for individuals with nonspecific symptoms, as it may mimic inflammatory bowel disease. The patient demonstrated a favorable response to anti-TB treatment, highlighting the significance of early detection and appropriate management of this uncommon disease. We believe this report contributes to the growing body of literature on extrapulmonary manifestations of TB.

## Introduction

Gastrointestinal tuberculosis (GI TB) is an uncommon form of extrapulmonary tuberculosis (EPTB), accounting for approximately 11 to 16% of EPTB cases [1]. TB is common in endemic regions such as Southeast Asia and Sub-Saharan Africa, and its incidence is increasing in developed countries, driven by factors such as immigration, HIV infection, and the use of immunosuppressive therapies [2]. GI TB can occur at any site in the gastrointestinal tract, with the ileocecal region involved in approximately 64% of cases, followed by the transverse colon, rectum, and ascending colon [3]. The pathogenesis of GI TB may involve hematogenous spread from active pulmonary disease, ingestion of infected sputum, direct spread from adjacent organs, or lymphatic dissemination from infected lymph nodes [4].

GI TB presentations often include symptoms such as abdominal pain, weight loss, fever, and changes in bowel movements, and only approximately 20% of GI TB cases have concurrent active pulmonary TB, making the diagnosis particularly challenging [5]. Patients may present with chronic abdominal pain accompanied by constitutional symptoms [6]. GI TB can frequently mimic other pathologies, including inflammatory bowel disease (IBD), particularly Crohn's disease, malignancy, or other causes of infectious enterocolitis. Despite advancements in diagnostic modalities, such as endoscopy, histopathology, and molecular testing, the presence of nonspecific symptoms and overlapping imaging findings frequently leads to delays in diagnosis and treatment, which can result in devastating consequences, including perforation, obstruction, or fistula formation [7].

## Case presentation

**Figure 1:**
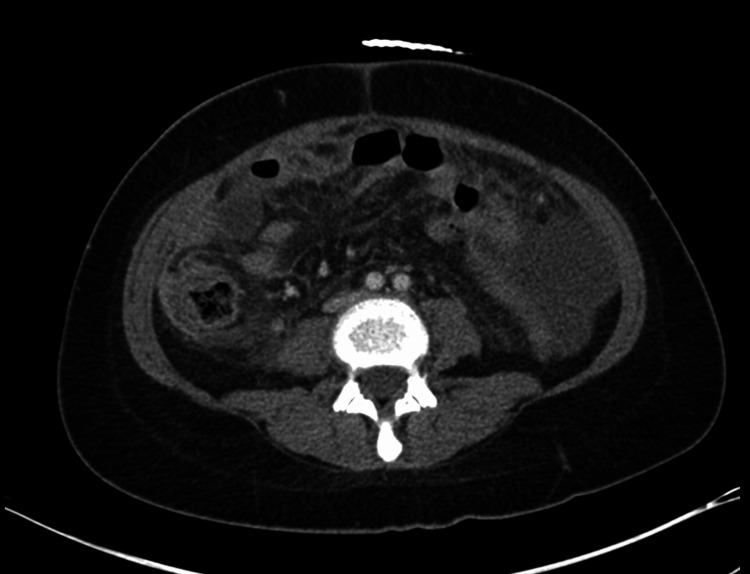
Axial images from CT abdomen pelvis with diffuse peritoneal thickening/enhancement consistent with peritonitis, prominent in R peritoneal reflection CT: computed tomography

**Figure 2:**
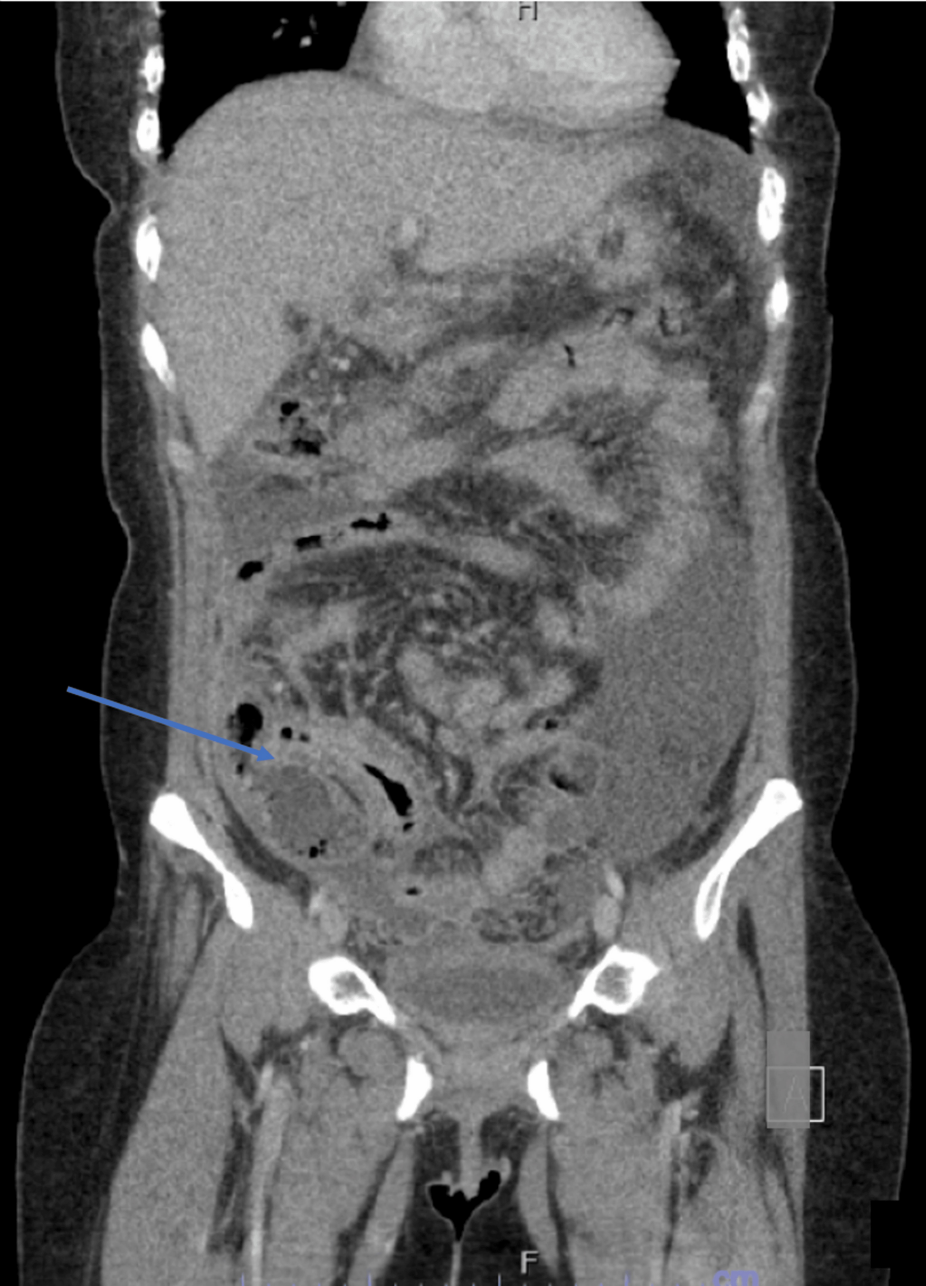
CT coronal images showing diffuse mural thickening and enhancement affecting the small bowel, cecum, and ascending colon CT: computed tomography

A 34-year-old Hispanic female with chronic lymphopenia and Trisomy 21 presented to the emergency department with worsening abdominal pain and nausea without vomiting. The patient reported an occasional cough with clear sputum and denied diarrhea. She had not recently traveled outside the country; her most recent trip had been to Mexico with her family several years ago. Blood work revealed normal liver tests, hypoalbuminemia with an albumin level of 1.6 g/dL (reference range: 3.5-4.8 g/dL), an elevated erythrocyte sedimentation rate of 81 mm/hr (reference range: 0-29 mm/hr), an elevated C-reactive protein of 272.2 mg/L (reference range: 0.0-7.0 mg/L), an elevated fecal calprotectin of 4820 mcg/g (reference range: 50-80 mcg/g), and negative infectious stool studies. Rheumatology recommended an evaluation for antiphospholipid syndrome and systemic lupus erythematosus. Testing yielded a low-positive ANA titer of 1:40 and a positive lupus anticoagulant, though the findings were otherwise not suggestive of an autoimmune process. An initial CT scan was performed in the emergency department, and the findings demonstrated marked diffuse mural thickening of the cecum and ascending colon with severe thickening of the terminal ileum; there was also associated peritoneal thickening and ascites (Figures 1-2).

**Figure 3:**
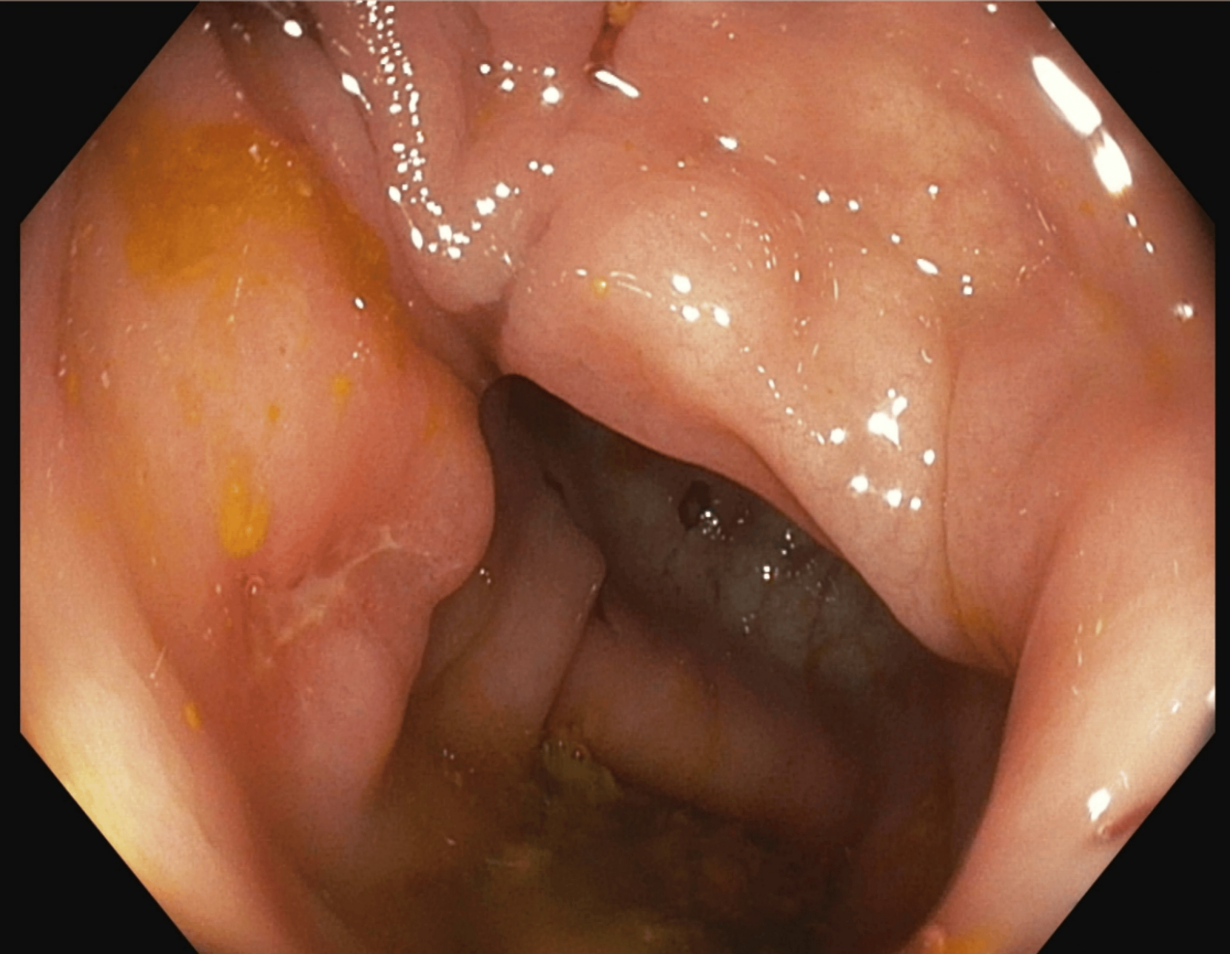
Endoscopy revealing cecal ulcer

**Figure 4:**
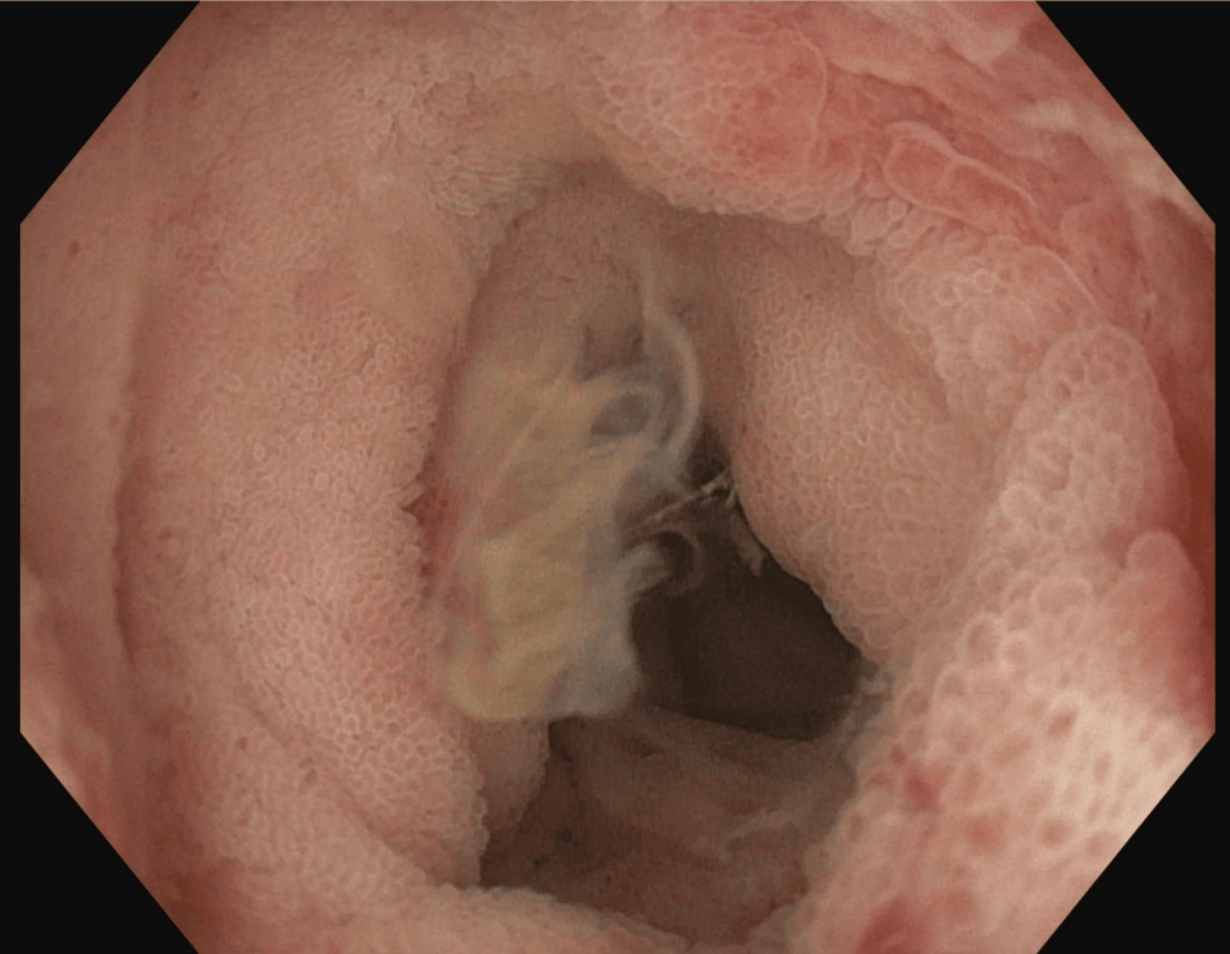
Terminal ileum inflammation with scattered ulcers

Systemic anticoagulation with a heparin drip was started for an incidentally identified left renal vein thrombus and a proximal left portal vein thrombus. Further testing did not suggest a hypercoagulable disorder. Empiric intravenous ceftriaxone and metronidazole were initiated for empiric coverage of intra-abdominal infection, given imaging findings of terminal ileitis. Diagnostic paracentesis for the patient’s new-onset ascites revealed high-protein ascites with a low serum ascites albumin gradient (SAAG), consistent with a non-portal hypertensive etiology, as may be seen in conditions such as tuberculous peritonitis and malignancy. Acid-fast bacilli (AFB) testing of the ascitic fluid was negative, and ascitic fluid cytology was negative for malignancy. CT of the chest showed moderate bilateral pleural effusions and a moderate pericardial effusion; diagnostic thoracentesis was deferred after discussion with Pulmonology, given the lack of a sufficient amount of fluid. In consultation with Gastroenterology, plans for an upper endoscopy with enteroscopy and colonoscopy were made to evaluate for GI TB, IBD, and malignancy. Endoscopic evaluation revealed terminal ileitis with several ulcers and a cecal ulcer, giving the impression of infectious enterocolitis or IBD (Figures 3-4).

**Figure 5:**
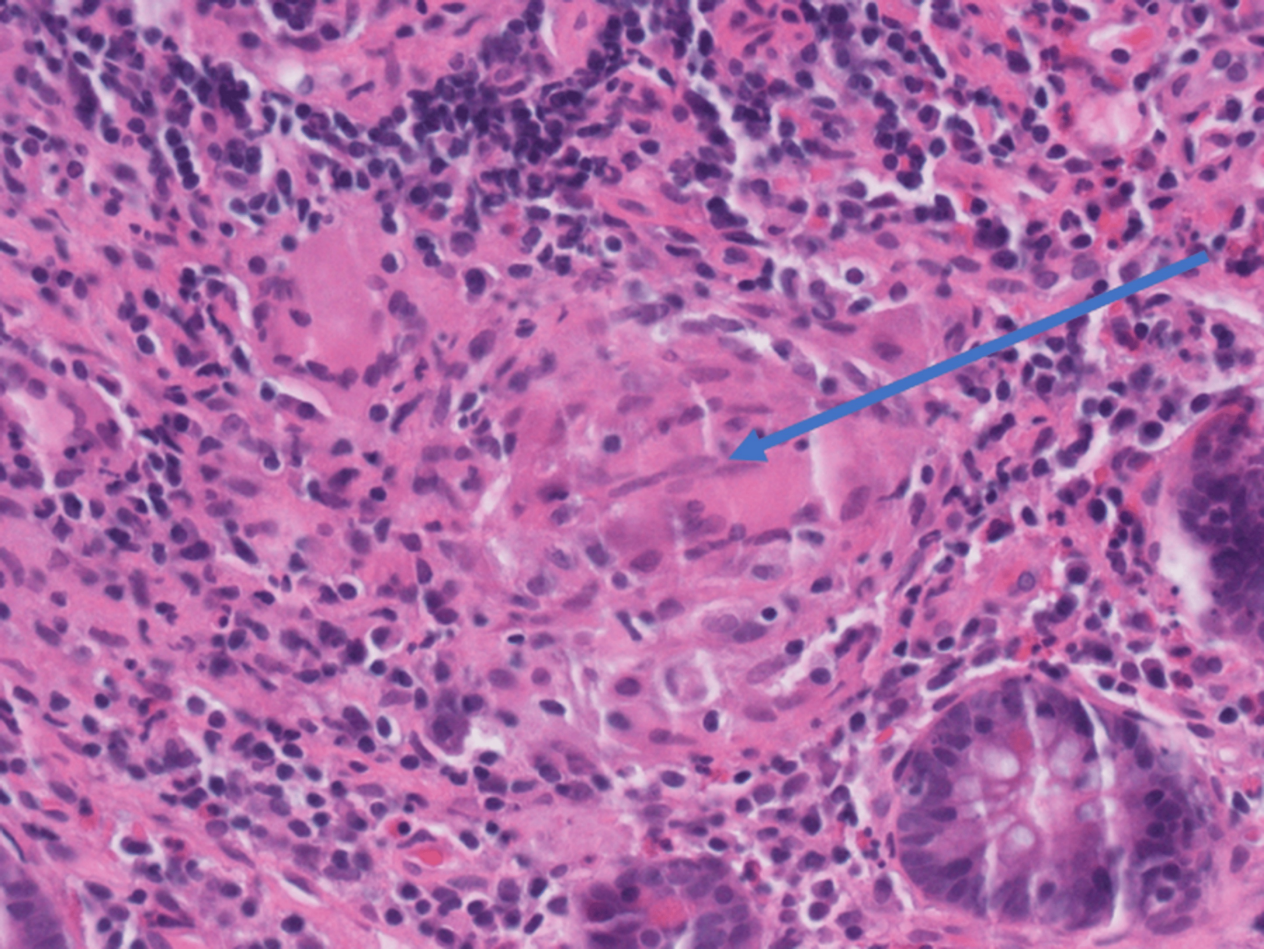
Histology findings from terminal ileum biopsy The non-necrotizing granuloma is located in the lamina propria. This H&E section (40x) reveals a non-necrotizing granuloma consisting of a few multinucleated giant cells and epithelioid histiocytes surrounded by lymphocytes

Biopsies of the cecal ulcer and terminal ileum were taken for AFB staining, fungal studies, CMV staining, and histologic review. Symptoms persisted despite initial empiric broad-spectrum antibiotics. An infectious workup, including testing for coccidioidomycosis, histoplasmosis, Strongyloides, and Entamoeba histolytica, was unrevealing. On day four, the polymerase chain reaction (PCR), specifically the GeneXpert *Mycobacterium tuberculosis*/rifampicin resistance (MTB/RIF) assay from an induced sputum sample, returned a positive result. Endoscopic biopsy later revealed noncaseating granulomatous ileitis of the terminal ileum and a cecal ulcer with focal cryptitis, mildly increased lymphocytes in the lamina propria, and a focus suggestive of a noncaseating granuloma, though AFB staining was negative (Figure 5).

In addition, given the positive MTB-PCR and high concern for TB, standard four-drug anti-TB therapy with rifampin 600 mg daily, isoniazid 300 mg daily with pyridoxine supplementation, pyrazinamide 1500 mg daily, and ethambutol 1200 mg daily (RIPE) was initiated. Levofloxacin 1000 mg daily was also initiated at the discretion of Infectious Diseases, with plans for further follow-up with the Department of Public Health for continued management. The patient showed significant clinical improvement; abdominal pain and nausea resolved following initiation of RIPE therapy. After completing seven days of isolation and confirmation that liver tests were stable, the patient was discharged on oral RIPE therapy with appropriate isolation precautions and scheduled follow-up with the Department of Public Health.

After discharge, one of three initial AFB sputum cultures ultimately grew Mycobacterium tuberculosis. Sensitivities revealed resistance to pyrazinamide. Pyrazinamide and ethambutol were subsequently discontinued due to elevations in liver tests. Rifampin was changed to rifabutin due to the initiation of rivaroxaban for thrombosis. The patient completed nearly nine months of TB treatment for gastrointestinal TB and pulmonary TB, but treatment was discontinued one week early due to drug-induced neutropenia. The patient experienced significant overall clinical improvement, and treatment was deemed adequate.

## Discussion

GI TB is a rather infrequent form of EPTB [1]. It often presents with nonspecific symptoms and may mimic Crohn’s disease, malignancies such as colorectal cancer, lymphoma, metastatic disease, or other infections. GI TB can also mimic other granulomatous diseases such as sarcoidosis. Presenting symptoms may include abdominal pain, fever, diarrhea, weight loss, constipation, and loss of appetite [6]. Severe complications can include intestinal obstruction, perforation, stricture, bleeding, or fistula, necessitating surgical intervention [8]. The terminal ileum and cecum are the most frequently affected sites due to their lymphatic supply and the stasis of intestinal contents [4]. Diagnosis is challenging, involving a combination of clinical suspicion, imaging, endoscopy, histology, and microbiology [6].

Although caseating granulomatous inflammation is a classic finding in TB, the presence of noncaseating granulomas in our case highlights the variability of histopathological findings. Obtaining biopsies revealing centrally necrotic granulomas, which are the most diagnostic, is often difficult due to sampling errors or their patchy distribution [9]. More recently, molecular diagnostics, including the GeneXpert MTB/RIF PCR assay, have improved the sensitivity (99.45%) and specificity (99.92%) of detecting *Mycobacterium tuberculosis* infection, especially in infections with very few bacilli [10]. These modalities aid in the diagnosis of TB in conjunction with conventional AFB stains and cultures, which often have low sensitivity in intestinal tissue samples [11]. In contrast to pulmonary TB, certain comorbidities, such as younger age or female sex, are associated with an increased risk of GI TB [8].

A comprehensive infectious workup is warranted to exclude alternative etiologies. CT and MRI are important imaging modalities for identifying characteristic features such as mural thickening, lymphadenopathy, and ascites. However, imaging alone is not definitive without histopathological or microbiological confirmation. Paracentesis can reveal low SAAG ascites, suggesting a non-portal hypertensive etiology; it is important to evaluate ascitic fluid cytology to rule out peritoneal carcinomatosis, as peritoneal imaging findings may overlap with those seen in gastrointestinal tuberculosis. Granulomas continue to play a key role in diagnosis. Insufficient necrotic material within granulomas can pose a challenge in the diagnostic process. The absence of caseation in this case emphasized the importance of correlating histological findings with clinical and microbiological data. Emerging research has also suggested a potential role for endoscopic ultrasound-guided fine-needle aspiration (EUS-FNA) in improving diagnostic accuracy, particularly in cases involving lymphadenopathy [12].

Typical management of GI TB involves anti-tuberculosis therapy with rifampin 600 mg daily, isoniazid 300 mg daily, pyrazinamide 1500 mg daily, and ethambutol 1200 mg daily for an initial two-month regimen, followed by four months of isoniazid and rifampin alone, in addition to close monitoring and supportive care [13]. Timely initiation is critical to prevent severe complications and to achieve favorable outcomes. It is important to maintain a high index of suspicion for GI TB in endemic areas and among high-risk populations such as immunosuppressed individuals. Further research is needed to improve diagnostic algorithms for GI TB and to expand access to advanced diagnostic tools, particularly in regions where TB remains endemic. Early detection and prompt initiation of standard RIPE therapy remain essential to prevent complications and ensure favorable patient outcomes.

## Conclusions

GI TB remains a clinical challenge, as it can mimic IBD, malignancy, and other granulomatous disorders. In patients who present with vague abdominal pain, weight loss, and constitutional symptoms, particularly when endoscopy or imaging reveals ileitis, colitis, peritoneal thickening or nodularity, or unexplained ascites, clinicians should broaden the differential diagnosis to include GI TB, especially in those with epidemiologic factors such as immigration from endemic regions, HIV infection, or immunosuppression. Risk factors for GI TB can include female sex and younger age, both of which were seen in this case. Mucosal biopsies can sometimes fail to identify the central caseous necrosis characteristic of TB. Infectious testing, such as AFB staining and cultures, QuantiFERON Gold, adenosine deaminase (ADA), and molecular tests, including MTB PCR testing, such as the GeneXpert MTB/RIF assay, can aid in confirmation.

Administering empiric RIPE therapy when there is a strong suspicion of TB can help prevent diagnostic delays, avoid unnecessary immunosuppression and surgery, and reduce the risk of obstruction or perforation. Ultimately, a multimodal approach including a detailed infectious workup, imaging, endoscopy with biopsy, targeted molecular testing, and timely antituberculous treatment is the most effective strategy for improving outcomes in this complex disease. This report adds an additional diagnostic case to the limited body of related literature, focusing on the complex diagnosis of GI TB. It provides another example of how the diagnosis was made, with the hope that future research can develop specific guidelines for establishing this diagnosis.
